# Statistical methods and resources for biomarker discovery using metabolomics

**DOI:** 10.1186/s12859-023-05383-0

**Published:** 2023-06-15

**Authors:** Najeha R. Anwardeen, Ilhame Diboun, Younes Mokrab, Asma A. Althani, Mohamed A. Elrayess

**Affiliations:** 1 Research and Graduate Studies, Biomedical Research Center, Qatar University, P.O. Box 2713, Doha, Qatar; 2 Department of Human Genetics, Sidra Medicine, Doha, Qatar; 3 QU Health, Qatar University, Doha, Qatar

**Keywords:** Metabolomics, Metabolomics tools, Statistical methods, Analytical workflow, Univariate, Multivariate

## Abstract

Metabolomics is a dynamic tool for elucidating biochemical changes in human health and disease. Metabolic profiles provide a close insight into physiological states and are highly volatile to genetic and environmental perturbations. Variation in metabolic profiles can inform mechanisms of pathology, providing potential biomarkers for diagnosis and assessment of the risk of contracting a disease. With the advancement of high-throughput technologies, large-scale metabolomics data sources have become abundant. As such, careful statistical analysis of intricate metabolomics data is essential for deriving relevant and robust results that can be deployed in real-life clinical settings. Multiple tools have been developed for both data analysis and interpretations. In this review, we survey statistical approaches and corresponding statistical tools that are available for discovery of biomarkers using metabolomics.

## Overview of metabolomics

The term metabolome was first coined in 1998 [1] and became widely established in the early 2000 [2]. Metabolomics profiling is a high-throughput technique that quantifies the levels of endogenous metabolites in a sample (biological fluids, tissues, etc.). [3]. The study of metabolites or metabolite profiling has been gaining popularity in the past decade, thanks to the recent advances in analytical platforms such as Fourier-Transform Infrared spectrometry (FT-IR), Nuclear magnetic resonance (NMR), mass spectrometry (MS) coupled to separation techniques such as gas-chromatography (GC–MS), liquid chromatography (LC–MS), Fourier Transform mass spectrometry (FT-MS), Ultra-high performance liquid chromatography (UPLC–MS), Capillary electrophoresis (CE–MS), Inductively coupled plasma (IPC–MS), Ion chromatography (IC–MS) [4] etc. Metabolites are key molecules in cellular functions. Many biological disturbances involve a cascade of metabolic changes, making metabolites close descriptors for the phenotype. There are two main analytical techniques that are used in the quantification of metabolites (in a cell, tissue, or body fluids): NMR and MS [5–7] through a process that can be untargeted or targeted. The former is a comprehensive technique measuring all metabolites in a sample without bias, including unknown chemical compounds. It is best suited for hypothesis-generating studies and leads to novel biomarker discovery, although the identification and categorisation of unknown compounds remains a great challenge. On the other hand, targeted metabolomics quantifies chemically known and annotated metabolites. Typically, the measured metabolites are labelled by comparing their masses to known compounds from spectral databases, which in addition to characteristic MS or NMR properties, also contain various information about nomenclature, compound concentrations, biological locations, enzyme and mutation data (see Table 1).

**Table 1:** Databases containing mass spectra data for metabolite annotation

Database	Comments	Source
Human Metabolome Database (HMDB 5.0)	217,920 known and 1,581,537 unknown compounds. Novel spectral data, physiological and pathological data, pathway data are available in a single platform [8]	https://hmdb.ca/
Golm Metabolome database	Dedicated to GC–MS technique. Contains custom libraries stored as mass spectra (MS) and retention time indices (RI) for metabolic profiling experiments and even observed mass spectral tags (MSTs) of unidentified metabolites	http://gmd.mpimp-golm.mpg.de/
Metlin	240,000 metabolite data is available as neutral or free acids, which enables single, batch, fragment, ion, neutral loss searches. High resolution of 72,000 MS/MS spectra is a key component of this database [9]	https://metlin.scripps.edu/landing_page.php?pgcontent
Massbank	MS database of high resolution spectral data with excellent structural searching methods. More than 41,000 spectra are available	https://massbank.eu/MassBank/
mzCloud	Freely accessible collection of mass spectra of endogenous and exogenous metabolites. Advanced searching capabilities enable finding metabolites that are not included in the library	https://www.mzcloud.org/

Since its introduction, metabolomics has been used in a wide range of applications such as health and disease biomarker and enzyme discoveries, food and nutrition, and plant biotechnology to name a few [10]. Metabolomics has proven to be a valuable tool in biomedical research, enabling the assessment of disturbances in biological systems caused by environmental factors, aiding in the diagnosis of diseases, and facilitating the identification of biomarkers. Biomarkers, short for, biological markers are objective indicators that provide information about cellular or organismal processes and can be used to characterize patients in a clinical setting [11]. Properties such as high specificity, sensitivity, repeatability, and clinical usefulness are necessary for a good biomarker. The process of biomarker validation entails in vitro and in vivo research followed by clinical trials in human cohorts. Biomarker discovery using metabolomics is considered to be a relatively improved method compared to traditional diagnostic approaches due to its sensitivity and specificity [12]. Metabolites have been found to be eligible molecular biomarkers in several studies; for instance, an untargeted metabolomics approach was used to show that non-alcoholic fatty liver diseases (NAFLD), featuring a range of severity levels from simple steatosis to complex hepatocellular carcinoma, are characterised each with a distinct metabolic profile [13, 14]. Furthermore, metabolomics have shown their potential in diagnosis and management in early screening of oral cancer [15], pancreatic cancer [16], and breast cancer [17]. Additionally, it was shown that recurrence can be monitored using metabolite biomarkers in various cancer patients [18–20]. Further to cancer, metabolomic studies have investigated potential biomarkers associated with fitness [21], telomere length [22], cardiovascular demand [23], steroid profile [24], etc. in elite athletes. Other studies evaluated biomarkers of metabolic diseases such as polycystic ovary syndrome [25], insulin resistance [26–29], and diabetes [30] (See Table 2 for examples of biomarkers from the mentioned studies). With the recent outbreak of COVID-19, emerging metabolomics data have provided insights into COVID-19 pathogenesis in patients with pre-existing chronic conditions such as diabetes, hypertension, hypothyroidism, etc. and revealed biomarkers linked to mechanisms of disease progression, severity, and side-effects of COVID-19 in affected individuals [31–38].

**Table 2:** Biomarker discoveries using metabolomics

Disease	Biomarker	Use	Ref
Esophageal squamous cell carcinoma (ESCC)	3′-UMP, palmitoleic acid, palmitaldehyde, and isobutyl decanoate	Disease recurrence	[39]
Hepatocellular carcinoma (HCC)	Leucine, valine, and tryptophan	Diagnostic biomarkers	[40]
Non- alcoholic fatty liver disease (NAFLD)	Glycocholic acid, Taurocholic acid, Phenylalanine, branched-chain amino-acids, Glutathione	Discrimination of steatosis, steatohepatitis and cirrhosis	[13]
Oral cancer	Pipecolate, Spermidine, Methionine, Tryptophan, Valine, Hypoxanthine, Trimethylamine N-oxide, Guanine, Guanosine, Taurine, Choline, Cadaverine, Threonine	Salivary biomarkers for oral cancer screening	[15]
Pancreatic cancer	1,5-Anhydo-d-glucitol	Diagnostic biomarker	[16]
Estrogen receptor negative breast cancer	Histidine, Glucose, Lactate, Tyrosine	Risk of disease recurrence	[17]
Colorectal cancer	Hexadecanedioic acid, 4-dodecylbenzenesulfonic acid, 2-pyrocatechuic acid, and Formylanthranilic acid	Screening and early detection using serum biomarkers	[41, 42]
Bladder cancer	Nɛ, Nɛ, Nɛ-trimethyllysine, N-acetyltryptophan, dopaquinone, leucine and hypoxanthine	Risk of disease recurrence	[20]
Sports related biomarkers	Glutamine, N-acetylglutamine, xanthine, beta-sitosterol, N2-acetyllysine, stearoyl-arachidonoyl-glycerol (18:0/20:4), N-acetylserine and 3–7-dimethylurate	Leukocyte telomere length prediction	[22]
	Arachidonic acid, branched-chain amino acids, plasminogens, phosphatidylcholines, phosphatidylethanolamines, Gamma-glutamyl amino acids and glutathione	Potential biomarker signatures for assessing health, performance, and recovery of elite athletes	[23]
	5alpha-androstan-3alpha,17alpha-diol monosulfate, androstenediol (3alpha, 17alpha) monosulfate and cortisol	Steroid profile difference in elite female players and non-athletes	[24]
Polycystic ovary syndrome	Hexosylceramide (d18:2/24:0), ceramide (d18.0/24.1) and serine	Predicting low birth weight	[25]
Insulin resistance and diabetes	Androsterone glucuronide, phenylalanine derivative, carboxyethylphenylalanine	Biomarkers associated with insulin resistance in lean/overweight females	[26]
	Glycerophosphoethanolamine, glycerophosphorylcholine and choline	Increased risk of obesity-associated insulin resistance	[27]
	Glutamate	Predictor of gestational diabetes mellitus	[30]
COVID-19	Tryptophan, kynurenine and 3-hydroxykynurenine	Prognostic markers	[33]
	A combination of d-fructose, citric acid and 2-palmitoyl-glycerol	Diagnostic biomarkers	[34]
	Palmitic (C16:0), docosapentaenoic (C22:5, DPA), and docosahexaenoic (C22:6, DHA) in diabetic patients palmitic, oleic (C18:1), and docosahexaenoic acids in hypertensive patients	Predicting disease progression	[36]
	Betaine and branched chain amino acids	Prognostic metabolic biomarkers of severity and mortality respectively	[37]

Early biochemical investigations, in the field of metabolomics, featured a low number of measured analytes to ease the interpretation of results [43, 44]. Today, information systems have matured tremendously, and many tools have been developed to assist in analysing and interpreting high throughput metabolomics data. With the continuous advances in instrumental techniques, adopting the correct statistical approach remains critical for proper interpretation and optimal utilization of data. The purpose of this review is to provide an overview of metabolomics data analysis in current research, with special emphasis on methods available for biomarker discovery in human disease.

## Metabolomics: analytical challenges and pre-processing

Like other omics fields, the workflow of metabolomics comprises of (i) Experimental design, (ii) Sample collection and preparation, (iii) Data retrieval/acquisition and pre-processing, and (iv) Data analysis and interpretation [45, 46]. Experimental design aids in tightening confidence intervals, minimising confounders and controlling the obvious sources of variation. Sample collection, preparation and data retrieval are the stages where systematic and random errors occur, although these can be controlled via strict work environment and protocol design to some extent [47]. It is during the pre-processing stage that the spectral data are converted to abundance of metabolites in each sample, a crucial link between raw data measurement and statistical analysis. Typical pre-processing steps include deconvolution, library-based identification, and alignment [48] which can be performed by a variety of analytical tools (refer to Table 3). For untargeted metabolomics, this step represents a major challenge due to the lack of spectra for the novel metabolites detected. However, methods to characterize the unknowns are being continuously explored. For example, Knowledge-guided multi-layer networks (KGMN), developed by Zhou et al., were used in untargeted metabolomics to enable global metabolite identification from knowns to unknowns by integrating knowledge-based metabolic reaction network, MS/MS similarity network as well as global peak correlation network [49]. Global network optimization approach, NetID, was recently developed by Chen et al. to annotate untargeted LC–MS data. NetID develops chemically meaningful peak-peak correlations, improves peak assignment accuracy, and creates a single network connecting most observed ion peaks, even for peaks missing MS spectra [50]. Statistical machine learning-based methods are geared towards the identification of unknowns based on feature similarity with the knowns: For instance, (MP-)IOKR [51], MetFrag [52] and CSI:FingerID[53] employ fragmentation trees to learn rules for subclustering of metabolites[52]. Methods like MetFusion [54] were developed to allow access to large spectral databases such as MassBank [55] to allow for improved optimization of predictive models.

**Table 3:** Quick view of sources for statistical analysis of metabolomics data

	MetaboAnalyst 5.0/MetaboAnalystR	Mzmine 3	Metabolyzer	PhenoMeNal	SECIMTools	Umetrics SIMCA	XCMS online / XCMS	MAIT	Omu (count data)	Specmine	pmartR	muma
Platforms	W/R	W	W	W	W	A	W/R	R	R	R	R	R
Ref	[56]	[57]	[58]	[59]	[60]	[61]	[62]	[63]	[64]	[65]	[66]	[67]
Pre-processing	✓	✓		✓	✓		✓	✓		✓	✓	✓
Imputation	✓	✓		✓	✓			✓		✓	✓	✓
Filtering				✓	✓	✓		✓		✓	✓	✓
Normalization	✓		✓	✓	✓	✓	✓	✓		✓	✓	✓
Univariate	✓	✓	✓	✓	✓		✓	✓	✓	✓	✓	✓
Multivariate	✓	✓	✓	✓	✓	✓	✓	✓	✓	✓	✓	✓
ROC analysis	✓		✓	✓	-	✓	✓	✓*		✓*	✓*	✓*

W: web-based, R: R programming language, A: Licensed application

*ROC analyses can be performed with base R functions while using these R packages

## Statistics in metabolomics

In addition to analysis challenges encountered with omics data such as high variable dimensionality and intercorrelation, metabolomics data are particularly prone to noise and can be influenced by environment factors, diet, exercise as well as sample handling and batch measurement. In addition, metabolomics data are characterised by a greater extent of data missingness which can compound multivariate analysis and classification techniques. As a consequence, careful application of appropriate statistical methods is required; otherwise, crucial information may get lost or false trends/models may be identified.

**Fig. 1:**
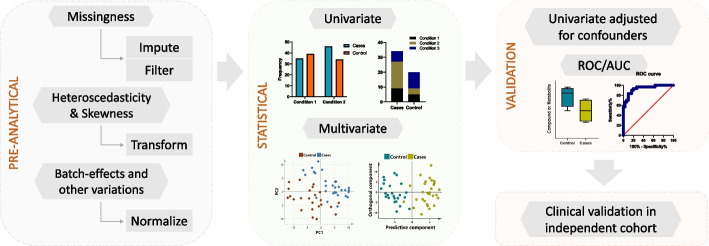
Simplified workflow of the statistical steps in metabolomics

The format of metabolomics data is typically a data matrix, with metabolite abundance and samples given in columns and rows or *vice-versa*. Even though metabolomics profiling is highly sought, there are no standard protocols established for the statistical analysis of the produced data. In this review, we discuss some of the widely adopted statistical approaches in recent studies. A simple schematic representation of the steps involved in metabolomics data analysis is depicted in Fig. 1.

### Pre-analytical steps

The metabolomics data matrix is prone to elevated metabolite missingness due to several reasons, most notably the inability to measure when metabolite levels are below the detection level as well as technical errors such as peak misalignment or metabolite structural instability. General statistical techniques for multiple imputation have been traditionally applied on metabolomics data but more tailored approaches that acknowledge the frequent non-random pattern of missingness in metabolomics have recently been developed: MetabImpute, an R package which can assess the missingness as completely or partially missing due to randomness and non-randomness (MCAR—missing completely at-random, MAR—missing at-random and MNAR—missing not-at-random) [68]. Indeed, there is no general opinion on the right filter percentage, but cut-offs have been traditionally chosen within the range of 20–50% of metabolite missingness [69, 70]. Imputation is crucial when multivariate techniques, including classification, are applied on metabolomics data.

Complex metabolomics data is heteroscedastic and right skewed and requires normalization. The go-to method for correcting the skewness is log-transformation [71]. Furthermore, filtering of overly heterogenous or bad quality samples is a good practise to avoid the propagation of errors throughout the dataset and can be achieved by means of multivariate techniques such as principal component analysis and clustering. Data normalization, based on aligning the median or more generally quantiles, is crucial to eliminate between-sample variation. It should be noted that using a wrong pre-analytical method to normalize/transform the data will result in poor results and may impact the ranks of relevant metabolites. Additionally, data points should only be removed if there are valid biological justifications for considering them as outliers. It is possible to produce a model that seems to work well by excluding difficult-to-model data points, but that is not actually representational of the real biological system.

## Statistical methods

Two main statistical approaches are available for metabolite differential level analysis: univariate and multivariate. Combination of both methodologies is common to metabolomic biomarker-based studies but this review shall focus on the advantages and increased power gained from multivariate analysis (MVA).

MVA is an essential part of metabolomics data analysis. Biological systems are not limited to single variable changes between healthy and diseased states. Investigation of system level changes is pivotal to deriving definitive conclusions about a certain condition and its potential biomarkers. MVA techniques incorporate all variables simultaneously and assess the relationships among them [72] as well as their joint contribution to the phenotype under study.

Unsupervised and supervised models of multivariate analysis are currently employed in metabolomics. One popular unsupervised technique is *Principal component analysis* (PCA) which identifies independent components in the data based on linear combinations of correlated features. Due to it unsupervised nature, PCA serves little purpose in biomarker discovery. PCA components are often fed into the univariate tests as a means for correcting for hidden unmeasured confounder effects. Moreover, PCA is often used as a checkpoint during QC to screen for outlier data points [73]. For example, when Al-Khelaifi et al. conducted PCA to obtain a global perspective of the data, they noted that PC1 captured the extent of haemolysis between the samples, while PC2 suggested effect of exercise. Incorporation of these components in the regression model greatly improved the detection of marker metabolites in association with the biological groups of interest to their study.

*PLS-DA or Partial Least Square Discriminant Analysis* [74] is a supervised MVA technique, that has been incorporated in numerous metabolomics studies for the discovery of biomarkers in different health conditions [75–77]. PLS-DA attempts for optimal break-down of predictor variable X to best explain the response variable Y [73, 78, 79]. An upgraded version of PLS-DA called OPLS-DA (“orthogonal” PLS-DA) has also gained popularity [80–84]. This model recapitulates the variance into parts that are predictive of the experimental groups and parts that are purely due to noise, also referred to as ‘orthogonal’ [85–87]. Therefore OPLS-DA creates decipherable models with ease in comparison to its previous version [88, 89]. Once the PLS/OPLS-DA model is built, the VIP (variable influence of projection) measure can be obtained for the metabolites based on their association with the identified predictive components. Certain studies use VIP > 1 as a threshold and select the metabolites for further analysis using linear regression models to correct for measured confounders.

*Support vector machines (SVM)* is a supervised machine-learning algorithm that can be used for regression and classification of non-linear data. SVM can detect non-linear relationships in the data that do not comply with the assumptions of PCA and OPLS, making it versatile. It identifies support vectors or samples on the margin between two classes to search for a maximum margin hyperplane. The use of kernels simplifies separation of classes for difficult cases by providing non-linear solution in the original space. SVM has different extensions for classification with overlapping groups, multi-class classification, regression, and specializations. Importantly, once the hyper-plane partition is found, feature importance values can be derived which can aid in biomarker discovery. However, basic SVM algorithms are not time efficient to tune complex separating hyperplanes as they do not take into account prior knowledge about probability of class-member. Despite often producing good results, faster and more stable methods can outperform SVM [90, 91]. A limitation of SVM is its restriction to binary classification. Alternative methods have been proposed to extend the use of SVM to multi-class problems, the models are built on breaking down the dataset into units of binary groups which causes oversimplification and may lead to uninformative models[92].

*Random forest (RF) is* a supervised machine learning method which is based on a decision tree algorithm. It is considered an excellent classifier for its ease of implementation, speed, stability robustness against overfitting and most importantly its ability to handle datasets with biased number of classes/groups [93]. Developed by Breiman, RF is a combination of decision trees, with each tree trained using a random subset of the data and input features. The algorithm uses a bootstrap sampling technique to select the data subsets. A simple RF with random features is created by randomly selecting a small group of input variables (with a fixed size) at each node to split on [94]. RF algorithm is highly adaptable to real-world datasets as it remains unaffected by scaling and normalization. However, a major challenge includes the requirement of excessive tuning of default parameters by the researcher to produce the best model, and eventual difficulty in visualizing the decision tree [95, 96]. Importantly, whilst classifying samples, RF performs a variable selection step which helps reduce the search space and aid in the process of pinpointing candidate metabolite biomarkers.

*Variational autoencoders* is an unsupervised deep learning method that operates by encoding input data into a non-linear, lower-dimensional latent space that can be used to reproduce the original data without loss of information. It has recently been advocated for use with metabolomics data to learn its transferable latent representations; which can help expose clusters of samples with specific metabolite levels [97].

The classification methods outlined in this review can be prioritised based on the research question and the characteristics of the data. SVM can handle binary and multinominal data with non-linear relationships between variables. RF, on the other hand, is compatible with continuous and categorical data and is used to create an ensemble of decision trees that can capture complex interaction between features, while being robust to outliers and normalization techniques. VAE is notably novel in the field of metabolomics and any added advantage to its use are yet to be shown.

OPLS/OPLS-DA is an excellent choice for small sized and highly correlated data with few groups of samples. It can explain most of the variation in the data by reducing the high dimensionality into predictive and orthogonal latent variables. It handles the missing values in the data efficiently and is robust to outliers [95]. One can argue that both RF and OPLS-DA methods are a good starting point for exploring metabolomics data due to their easiness of use and interpretability. Table 4 provides an overview of the methods, their strengths and weaknesses to be considered in metabolomics data analysis.

**Table 4:** Synopsis of popular statistical methods for metabolomics studies

	Methods	Strengths	Limitations
Univariate	T test Mann Whitney Chi-square ANOVA Kruskal Wallis	Straightforward application Easy to interpret the results	Requires prior knowledge of data No information about inter-variable relationships that is crucial in a biological set-up Outliers cannot be determined
	Multiple linear regression with Bonferroni correction (with one explanatory variable)	Easy to apply and interpret	Significance level affected by sample size Does not account for intercorrelation
	Multiple linear regression with false discovery rate (with one explanatory variable)	Easy to use and interpret Preferred over Bonferroni method	Increases the number of false negatives
Multivariate	Principle component analysis	Effective in variable reduction Uses the complete collected data Easy to manage complex data Focuses on the inter-variable relationships Requires no prior knowledge of data	No clarity on how to rank the metabolites Biological interpretation may be challenging
	Partial least square discriminant analysis Orthogonal partial least square discriminant analysis	Dimensional reduction to comprehensible level No data wastage Shows relationship between variables, apt in a biological setting Handles large, complex data	Prior knowledge of data required Over-fitting issues No significance level of the most important metabolites Abundant variables mask the effect of lesser abundant variables Cross-validation steps required to predict accuracy of model
	Random Forest, SVM and other ML methods	Handles complex data Robust to outliers Finds complex relationships between metabolites and between metabolite and other factors	Excessive tuning may be required to retrieve best model Less efficient for truly linear data Does not provide metabolite selection

It is important to note that classification, prediction and biomarker discovery methods for metabolomics data extend to other models including logistic regression models, LASSO, CCPLS, ASCA + and APCA + (extension of ANOVA to multivariate classes) [98], multivariate curve resolution (MCR), neural networks, Gaussian mixture modelling etc. More details about these methods and how they have been deployed in the field of metabolomics can be found in [99–103].

### Validation of model performance

Several metrices exist for assessment of model performance. With OPLS and PLS models, typical measures are R^2^ which captures the goodness of fit, and the *Q*^2^ that computes the predictive ability of the model, defined as the congruence of cross-validation of predicted data with the original data. OPLS further splits *R*^2^*X* into *R*^2^Xp and *R*^2^Xo which respectively measure the explained sum of squared of the Y-predictive and Y-uncorrelated parts of *X*. [104]. *Q*^2^ > 0.4 provides a satisfactory predictability of the model [105, 106]. *Q*^2^ and *R*^2^ values that are closer to 1 ensure a reliable model, while large discrepancy between the two scores depict an unreliable model [107]. Permutation tests are used to estimate Q^2^ and provide a possibility of calculating significance (*p*-values) for these MV models [108–110].

Brier score is another CV procedure that measures the accuracy of binary outcome predictions by calculating the squared difference between the actual outcome and predicted probability. A perfect model has a score of 0 and a non-informative model has a score of 0.25[111]. Harrell’s C-index is also a performance measure used with survival analyses. The index is driven by Kendall's tau statistic, depends on the censoring distribution, and considers the rankings of pairings of subjects in the data. The index ranges from 0 to 1 (indicating worst to best performance) and a value of 0.6 or higher is acceptable for clinical datasets [112].

The receiver operating characteristics (ROC) curve analysis assesses the specificity and sensitivity of a potential biomarker by plotting the true positive rate (y axis) as a function of the false positive rate (*x* axis). It produces the area under the curve (AUC) measure that indicates the ability of a biomarker to distinguish between two study groups. Multivariate receiver operating characteristic analysis (MultiROC) [113] is an extension of ROC analysis that allows for different combinations of biomarkers to be clinically explored [114, 115] and is compatible with the inherent nature of multivariate classifiers such as PLS/OPLS-DA models.

There are other cross-validation procedures employed in predictive analysis such as leave-n-out, Monte Carlo cross-validation (MCCV), corrected-MCCV (CMCCV) etc. For detailed information, readers are referred to Sammut et al. and Xu et al. [116, 117].

The metrices outlined above have been instrumental in assessing the performance of MV classification methods to ensure validity and reliability of the results. For example, a study by Chen et al. compared four classifiers, PCA, SVM, LDA and RF using several methods including cross-validation, *R*^2^/*Q*^2^ plot, ROC curve and Pearson corelation. RF was found to be associated with better performance with respect to sample classification and biomarker selection [118].

## Tools available for the statistical analysis of metabolomics data

Several tools are available for data analysis in metabolomics. The tools required for highly intricate metabolomics data analysis should be able to handle the large data size, perform pre-processing steps adequately, conduct statistical methods to identify significantly different metabolites, and provide striking visualization techniques such as heatmaps, correlation and pathway networks. We intend to cover some of the widely used tools that provide data pre-processing, univariate and multivariate methodologies used for biomarker discovery. Table 3 provides a quick view of the methods available in the tools discussed below.

(i) MetaboAnalyst: Extensive web-based toolkit for complete data analysis of metabolomics data. It provides multiple statistical workflows for one-factor, two-factor/timeseries, meta-analysis data formats, which include univariate (t-tests, ANOVA) and multivariate (PCA, PLS-DA, OPLS-DA). The latest version (MetaboAnalyst 5.0) is user-friendly compared to its predecessor. It contains a biomarker discovery option using ROC analyses with straightforward data input and user-defined options for pre-processing steps and normalization. This web-based platform has been utilized in various studies for biomarker identification due to its amenable nature [119–123].

(ii) MZmine 3:

Built on the success and popularity of MZmine 2, MZmine 3 is an open-source platform for data pre-processing and analyses with LC-MS in mind. The updated version has focused on improving the user-friendly graphics with the original eight modules [124].

(iii) MetaboLyzer:

It is a command line interface (CLI) providing general as well as metabolomics-suited statistical analysis and data visualization [125]. Integration with small-metabolite databases such as HMDB, KEGG, BioCyc and LipidMaps allows for ion identification and relevant data analysis. We would argue that it is more appropriate for expert-level bioinformatician in terms of user-friendliness.

(iv) PhenoMeNal:

To our knowledge, a comprehensive and unmatched tool that brings metabolomics to cloud computing after Galaxy. Ongoing immense data generation requires cloud-based tools to reduce the load on personal or workplace environment by storing the data onto cloud space. Data analysis tools are tested and stored as Docker containers [126]. PhenoMeNal has successfully developed sophisticated data analysis workflows, which reduces the burden on the researcher.

(v) SECIMTools (SouthEast Center for Integrated Metabolomics):

Designed to complement both the previous Galaxy metabolomics tools, Galaxy-M and Workflow4metabolomics, SECIMtools begins with features which follows quality control (QC) and advanced statistical assessment. It has four major functionalities: data pre-processing, QC, data analysis and utilities [127]. A guide to use the galaxy interface of SECIMTools can be found here. [https://ctsi-secim.sites.medinfo.ufl.edu/files/2015/08/7_7_2015_Galaxy_UserGuide.pdf]

(vi) SIMCA®

By Sartorius AG, SIMCA is the tool of choice for multivariate analysis by many studies [108]. It is user-friendly, with multiple interactive visualization methods, has the ability to fit models that best suit the data at hand, perform ROC analysis, analyse multiple datasheets, to name a few. For metabolomics, investigation of metabolites with significantly different abundances, metabolite pathways (if present in the datasheet) associated with experimental groups, examining relationship between variables and quick identification of potential biomarkers are relatively easy for non-programmers. SIMCA contains in-built cross validation steps that provide the predictive ability of the model. Although this tool is not suited to univariate analysis and is not in an open-source format and requires license purchase prior to use.

(vii) R (R foundation for statistical computing, Vienna, Austria) [128] packages for metabolomics:

For statisticians who are well-versed in programming languages, R is the best option for metabolomics data analysis as it provides a more flexible work environment as opposed to rigid online tools with limited user-defined options. There are several packages for normalization, imputation, univariate hypothesis testing, multivariate exploratory analysis in R.

(a) XCMS.

R based powerful tool for processing of LC-MS data using retention time correction, peak identification and matching to derive necessary information. It can be combined with base R functions to perform all statistical methods for a comprehensive data analysis.

(b) MetaboAnalystR.

Corresponding R package of web-based MetaboAnalyst, with more adjustable programming feature to enable autonomy of metabolomics data analysis.

(c) MAIT (Metabolite Automatic Identification Toolkit):

Provides a comprehensive end-to-end analysis for LC-MS data. Although it is more suited to peak identification and annotation. Parametric and non-parametric univariate statistical tools and multivariate analyses such as PLS-DA are available with user defined grouping option [63].

(d) Omu.

Performs simple t-tests, ANOVA, PCA and combine functional information and the associated gene names of the metabolites in the dataset using KEGG. It was developed for inexperienced R users to analyze metabolite count data. The input format should contain KEGG IDs to process the data. The package contains multiple visualization techniques such boxplots, heatmaps, volcano plots etc. [64].

(e) Specmine.

Multi-level analysis is available in this package, which includes pre-processing, metabolite annotation, uni- and multivariate analyses, ML (machine learning) and selection of significant features [65].

(f) pmartR.

Quality control processing, statistical analysis of metabolomics, lipidomis and proteomics data can be performed using pmartR. Analyses such as transformation, normalization, simple univariate and summarising PCA and correlation analyses are available [66].

(g) muma.

Built with non-programmers in mind, muma provides user-friendly stepwise univariate and multivariate analysis via R program. Data pre-processing, imputation, data exploration through various visualizations and statistical analysis are available in this package [67].

## Limitations of statistics in biomarker discovery

Biomarkers are measured indicators of biological and/or pathogenic processes, or response to therapies [11]. Metabolite biomarkers are quantified at a cheaper rate compared to other types of biomarkers [129]. There is certainly a rapid increase in the number of metabolite biomarkers discovered due to improvements in the analytical procedures but are not in practical use due to limitations in experimental design, statistical rigor, and efficacy [130, 131]. Biomarkers in clinical practice should be easy to quantify and should bring value in relation to early detection of disease, improvement in treatment outcomes, reduction in the reliance on expensive treatment options, or decrease in disease-related fatalities. Unfortunately, appropriate biomarkers with appreciable specificity and sensitivity are hard to come by. Using the combinatorial capacity of a variety of distinct biomarkers is one possibility to improve the overall specificity [132, 133]. Present-day metabolomics have substantially benefited from upgraded study design that contributed to the decrease in the demographic differences and sources of bias. This approach has been applied to all sorts of study designs such as interventional, observational, and with multi-tiers. Study enrolment with balanced demographic attributes under a multi-cohort setting should have sufficient sample size to comply with the requirements for adequate statistical power [134]. Improvised prospective trials are required to verify biomarkers’ ability to detect physiological changes before onset of phenotype. Validation of biomarkers has been carried out in small, unbridled trials so far [135]. However, large scale validation remains inadequate leading to very few metabolomics biomarkers finding their way to clinical practice [136, 137]. More insights on ways in which metabolomics research can be advanced to meet the challenges of biomarker discovery can be in found in Poste et al. [136].

## Conclusion

This mini review has introduced the user to standard methodologies with easy-to-use tools for analysis of metabolomics datasets and biomarker discovery. Metabolite biomarkers are constantly growing interest in the omics field as they depict a phenotype as close to accurate as possible from the physiological or pathological state. In the future, we expect the evolution of existing statistical methods to provide even deeper insights into metabolite biomarkers from the larger perspective of systems’ biology and precision medicine. In this context, biomarkers identified using multi-omics techniques can broaden the scope of individualized treatment plans by providing markers for patient stratification, early diagnosis, prediction, and progression monitoring, etc. To this end, advanced statistical and machine learning methods are being developed to provide effective approaches for multi-omics data integration [138]. Aligning the biological information from multi-level omics analysis has the advantage of reducing noise and provides an extra level of biomarker validation. More importantly, integration with genotype data can help distinguish biomarkers associated with causal effects as opposed to those of secondary nature, that occur because of the disease or pathology of interest as well as those contributed by the environment. Methods for stratification of patients into homogeneous groups with unified analyte levels, such as supervised biclustering [132, 133], have been recently applied in the field of transcriptomics and offer an interesting opportunity for metabolomics to embark in the field of precision medicine.

In parallel to technological advancement, progress in computational and statistical analysis is also required to tackle some of the remaining limitations in the field of metabolomics; notably with regard to annotation/identification of unknown compounds with untargeted metabolomics. Machine learning approaches are of great value in this respect and can offer better performance with improved and more accurate information on compound masses, retention time, fragment mass spectra, and isotopic properties [134].

It should be noted that all statistical methods incorporated in the field of omics are simply hypothesis creators, essentially shortening a seemingly limitless list of metabolites to a manageable set whose properties and merits should be evaluated by downstream experimental work. Standardization of validation protocols including replication and experimental validation in animal models is essential for metabolite biomarkers to make their way to pre-clinical settings.

## Data Availability

Not applicable.

## Abbreviations

AUC: Area under the curve
CE-MS: Capillary electrophoresis–mass spectrometry
CMCCV: Corrected- Monte Carlo cross-validation
FT-IR: Fourier-transform infrared
FT–MS: Fourier transform–mass spectrometry
GC–MS: Gas chromatography–mass spectrometry
IPC-MS: Inductively coupled plasma mass spectrometry
IC–MS: Ion chromatography–mass spectrometry
KGMN: Knowledge-guided multi-layer networks
KEGG: Kyoto encyclopedia of genes and genomes
LC–MS: Liquid chromatography–mass spectrometry
LDA: Linear dimension analysis
MCCV: Monte Carlo cross-validation (MCCV)
NMR: Nuclear magnetic resonance
ROC: Receiver operating characteristics
UPLC–MS: Ultra-high performance liquid chromatography–mass spectrometry
